# Delayed remnant kidney function recovery is less observed in living donors who receive an analgesic, intrathecal morphine block in laparoscopic nephrectomy for kidney transplantation: a propensity score-matched analysis

**DOI:** 10.1186/s12871-020-01081-z

**Published:** 2020-07-06

**Authors:** Jaesik Park, Minju Kim, Yong Hyun Park, Misun Park, Jung-Woo Shim, Hyung Mook Lee, Yong-Suk Kim, Young Eun Moon, Sang Hyun Hong, Min Suk Chae

**Affiliations:** 1 Department of Anesthesiology and Pain Medicine, Seoul St. Mary’s Hospital, College of Medicine, The Catholic University of Korea, 222, Banpo-daero, Seocho-gu, Seoul, 06591 Republic of Korea; 2 Department of Urology, Seoul St. Mary’s Hospital, College of Medicine, The Catholic University of Korea, Seoul, Republic of Korea; 3 Department of Biostatistics, Clinical Research Coordinating Center, Catholic Medical Center, The Catholic University of Korea, Seoul, South Korea

**Keywords:** Intrathecal morphine block, Remnant kidney function, Laparoscopic donor nephrectomy

## Abstract

**Background:**

This study analyzed remnant kidney function recovery in living donors after laparoscopic nephrectomy to establish a risk stratification model for delayed recovery and further investigated clinically modifiable factors.

**Patients and methods:**

This retrospective study included 366 adult living donors who underwent elective donation surgery between January 2017 and November 2019 at our hospital. ITMB was included as an analgesic component in the living donor strategy for early postoperative pain relief from November 2018 to November 2019 (*n* = 116). Kidney function was quantified based on the estimated glomerular filtration rate (eGFR), and delayed functional recovery of remnant kidney was defined as eGFR < 60 mL/min/1.73 m^2^ on postoperative day (POD) 1 (*n* = 240).

**Results:**

Multivariable analyses revealed that lower risk for development of eGFR < 60 mL/min/1.73 m^2^ on POD 1 was associated with ITMB, female sex, younger age, and higher amount of hourly fluid infusion (area under the receiver operating characteristic curve = 0.783; 95% confidence interval = 0.734–0.832; *p* < 0.001). Propensity score (PS)-matching analyses showed that prevalence rates of eGFR < 60 mL/min/1.73 m^2^ on PODs 1 and 7 were higher in the non-ITMB group than in the ITMB group. ITMB adjusted for PS was significantly associated with lower risk for development of eGFR < 60 mL/min/1.73 m^2^ on POD 1 in PS-matched living donors. No living donors exhibited severe remnant kidney dysfunction and/or required renal replacement therapy at POD 7.

**Conclusions:**

We found an association between the analgesic impact of ITMB and better functional recovery of remnant kidney in living kidney donors. In addition, we propose a stratification model that predicts delayed functional recovery of remnant kidney in living donors: male sex, older age, non-ITMB, and lower hourly fluid infusion rate.

## Background

Kidney transplantation (KT) is a preferred definitive cure for patients with end-stage kidney disease, as it is associated with better survival rate, and improved quality of life, compared to renal replacement therapy methods (e.g., dialysis) [1]. The substantial increase in prevalence of patients requiring renal replacement therapy has augmented the demand for grafts, and the kidney graft survival rates from deceased donors have been shown to be significantly inferior to those from living related or unrelated donors. This may be due to the very short cold ischemic time and better-functioning nephron mass of kidneys from healthy living donors. Thus, living donor KT has emerged as an effective clinical option to resolve graft shortage [2, 3]. Although the safety of living donor KT has been established, living donors undergoing nephrectomy may have long-term risks of cardiovascular events and/or progression to remnant kidney dysfunction [4].

Compensation and recovery of remnant kidney function after donation surgery require a baseline level of clinical suitability. Perioperative contributors for delayed recovery of remnant kidney function include hypertension, diabetes mellitus (DM), history of smoking, and obesity [5]. However, few studies have investigated the role of analgesic treatment, which might affect the sympathetic stress response and influence the degree of recovery in remnant kidney function. Kidney function can be compromised by many factors, including hypoxic and inflammatory damage, hormonal alterations (including in cortisol, catecholamine, anti-diuretic hormone, and renin-angiotensin-aldosterone), and inadequate repair mechanisms. These deleterious effects seem to be triggered and activated by surgical nociceptive/noxious stimuli, and are ultimately associated with decreased intra- and postoperative vascular flow [6–8]. Healthy living donors undergoing nephrectomy may be more susceptible to postoperative pain than ill patients undergoing nephrectomy. Because appropriate pain control is recommended after donation, intrathecal morphine block (ITMB) is an acceptable treatment for significantly reducing the severity of postoperative pain on post-operative day (POD) 1 [9–12].

This study primarily assessed remnant kidney function recovery in living donors undergoing laparoscopic nephrectomy to establish a risk stratification model for delayed recovery, and further investigated risk factors that were clinically modifiable, including ITMB.

## Methods

### Ethical considerations

The study protocol was approved by the Institutional Review Board of Seoul, St. Mary’s Hospital Ethics Committee (approval no. KC19RISI0911; December 26, 2019). The study was performed in accordance with the principles of the Declaration of Helsinki. The requirement for informed consent was waived because of the retrospective nature of the study.

### Study population

**Fig. 1:**
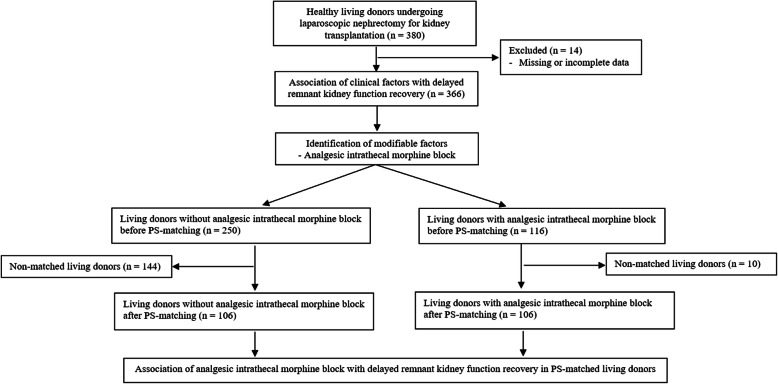
Flow diagram of the study

Electronic medical records were retrospectively reviewed for 380 living donors (> 19 years of age) who underwent elective laparoscopic nephrectomy for KT between January 2017 and November 2019 at Seoul St. Mary’s Hospital. Using the clinical practice guideline [13], a multidisciplinary consult team regularly assessed the clinical and psychological condition of the living kidney donors. Donors in our study population had American Society of Anesthesiologists physical status I or II, a tolerable estimated glomerular filtration (eGFR) rate (i.e., ≥60 mL/min/1.73 m^2^), and no evidence of a pathological renal lesion on abdominal computed tomography (CT). Because of missing or incomplete data, 14 living donors were excluded; finally, 366 adult living donors were enrolled in this study. A study flow chart is shown in Fig. 1.

### Surgery and anesthesia

Laparoscopic living donor nephrectomy was performed by an experienced urologic surgeon (Y.H.P.), using a method described in detail elsewhere [14]. Experienced attending anesthesiologists provided balanced anesthesia, with electrocardiography and standard vital monitoring of systolic blood pressure (SBP) and diastolic blood pressure (DBP), heart rate (HR), O_2_ saturation, body temperature, and capnography. Induction of anesthesia was performed using 1–2 mg/kg propofol (Fresenius Kabi, Bad Homburg, Germany) and 0.6 mg/kg rocuronium (Merck Sharp & Dohme Corp., Kenilworth, NJ, USA); maintenance of anesthesia was then performed using 2.0–6.0% desflurane (Baxter, Deerfield, IL, USA) with medical air/oxygen. Remifentanil (Hanlim Pharm. Co., Ltd., Seoul, Republic of Korea) was administered at a rate of 0.1–0.5 μg/kg/min, as appropriate. The Bispectral Index™ measurement (Medtronic, Minneapolis, MN, USA) was maintained between 40 and 50 to assure suitable hypnotic depth. Rocuronium was routinely infused under train-of-four monitoring (> one twitch). End-tidal CO_2_ was set between 30 and 40 mmHg through adjustment of the ventilator mode. Liberal fluid was administered during surgery, and mannitol (25 g) was administered immediately before ligation of the renal artery.

All living donors were administered postoperative intravenous (IV) patient-control analgesia (IV-PCA) (AutoMed 3200; Acemedical, Seoul, Republic of Korea), which included 1000 μg fentanyl (Dai Han Pharm. Co., Ltd., Seoul, Republic of Korea), 90 mg ketorolac (Hanmi Pharm. Co., Ltd., Seoul, Republic of Korea), which was supplied as an analgesic adjuvant at a low infusion rate to reduce the opioid requirement and thus avoid serious side effects (such as nephrotoxicity and bleeding) [15–18], and 0.3 mg ramosetron as an anti-emetic adjuvant (Naseron; Boryung Co., Ltd., Seoul, Republic of Korea). The IV-PCA program consisted of a 1-mL bolus injection and a 1-mL basal infusion of the IV-PCA solution, with a lockout time of 10 min. When living donors experienced acute severe postoperative pain (pain score ≥ 7 on a numeric rating scale [NRS]), rescue IV drugs for pain relief were administered based on preferences and discretion of the attending physicians in the post-anesthesia care unit and ward.

### ITMB intervention

Depending on the condition of healthy living donors, pain tolerance may be low [19, 20]. Therefore, ITMB, which is recognized as a safe and effective method of pain relief for living donors [12, 21], was included as an analgesic component in the living donor treatment strategy for early postoperative pain relief from November 2018 to November 2019. The day before donation surgery, informed consent for ITMB intervention was obtained from the living donors. Living donors who preferred to receive no ITMB intervention were provided with conventional analgesic service, including IV-PCA and rescue IV analgesic drugs.

To allow immediate identification of any nerve injury during the intrathecal practice performed before the induction of general anesthesia, living donors were provided no sedative medication in the operating room. Under standard vital sign monitoring, the living donors were positioned in the right or left lateral decubitus position, and the skin over the lumbar region was cleaned with chlorhexidine and draped. The donors received 0.2 mg (0.2 mL) intrathecal morphine sulfate (BCWORLD Pharm. Co., Ltd., Seoul, Republic of Korea) with normal saline (0.8 mL) using a sterile 25G Quincke type-spinal needle (TAE-CHANG Industrial Co., Ltd., Chungcheongnam-do, Republic of Korea) between lumbar vertebrae 3 and 4. Morphine sulfate and normal saline (total 1.0 mL) were administered as a single injection after cerebrospinal fluid had been obtained.

### Estimated glomerular filtration rate

Kidney function was quantified based on the eGFR, calculated using the Modification of Diet in Renal Disease formula: eGFR = 175 × standardized serum creatinine^-1.154^ × age^-0.203^ × 1.212 (if black) × 0.742 (if female) [22]. The baseline eGFR was estimated on the day before surgery, and serial eGFRs were measured on PODs 1 and 7. Based on the eGFR [23], the degree of kidney function was classified as normal function (eGFR ≥90 mL/min/1.73 m^2^); mild dysfunction (eGFR 89–60 mL/min/1.73 m^2^); and moderate dysfunction (eGFR 59–30 mL/min/1.73 m^2^). In our study, delayed functional recovery of remnant kidney was defined as eGFR < 60 mL/min/1.73 m^2^ on POD 1.

### Clinical variables

Preoperative findings included sex, age, body mass index (BMI) (divided into ≥25 kg/m^2^ [overweight] and < 25 kg/m^2^ [normal weight]) [24], and hypertension, which was controlled to achieve the blood pressure goal (which is usually < 140/90 mmHg, but is < 130/80 mmHg for those with diabetes or chronic kidney disease) with or without anti-hypertensive drugs [25], eGFR, laboratory variables (white blood cell count, hemoglobin, platelet count, glucose, albumin, sodium, potassium, chloride, international normalized ratio, and activated partial thrombin time), and remnant kidney volume estimated using abdominal CT images and volume software (AW VolumeShare 4; General Electric Healthcare, Chicago, IL, USA). Intraoperative findings included a time effect, thus the serial order of the living donors from the first (no. 1) to the most recent (no. 366), ITMB status, total surgery duration, average vital signs (i.e., SBP, DBP, HR, and body temperature), hourly fluid infusion, hourly urine output, and total blood loss. Postoperative findings included eGFR, peak NRS, cumulative IV-PCA consumption, peak hemodynamic parameters (i.e., SBP, DBP, and HR), laboratory variables (i.e., white blood cell count, hemoglobin, platelet count, sodium, potassium, and chloride), ITMB-associated complications (i.e., intrathecal site infection, post-dural puncture headache, lower limb numbness, respiratory depression, and bleeding), and surgical complications assessed using the Clavien-Dindo classification [26].

### Statistical analyses

The normal distribution of continuous findings was estimated using the Shapiro–Wilk test. Continuous data are expressed as means ± standard deviations (SDs) or medians (interquartile ranges). Categorical data are expressed as numbers and proportions. Perioperative findings were compared using the Mann–Whitney *U* test and *χ*^2^ test or Fisher’s exact test, as appropriate. The associations of pre- and intraoperative findings with delayed functional recovery of remnant kidney were evaluated by univariable and multivariable logistic regression analyses. Potentially significant findings (*p* < 0.1) in univariable analyses were entered into the multivariable analysis. The accuracy of the risk stratification model for delayed functional recovery of remnant kidney was estimated according to the area under the receiver operating characteristic curve. Preoperative and intraoperative findings in the non-ITMB and ITMB groups were assessed by propensity score (PS)-matching analysis. PS-matching analysis was performed to reduce the effect of potential confounding findings on intergroup differences according to the ITMB intervention. PSs were derived to match living donors at a 1:1 ratio using greedy matching algorithms without replacement. After the PS-matching had been completed, we assessed the balance in baseline covariates through paired *t*-tests and McNemar’s tests, as appropriate for continuous and categorical variables. The association of ITMB intervention with delayed functional recovery of remnant kidney was evaluated by multivariable logistic regression analyses with PS adjustment. The values are expressed as odds ratios with 95% confidence intervals (CIs). All tests were two sided, and *p* < 0.05 was considered to indicate statistical significance. All statistical analyses were performed using R software version 2.10.1 (R Foundation for Statistical Computing, Vienna, Austria) and SPSS for Windows (ver. 24.0; IBM Corp., Armonk, NY, USA).

## Results

### Perioperative baseline findings in living donors undergoing laparoscopic nephrectomy

Table 1 shows the pre-, intra-, and postoperative patient characteristics. No living donors had a history of DM. On PODs 1 and 7, there were no living donors with eGFR < 30 mL/min/1.73 m^2^ and/or requiring renal replacement therapy.

**Table 1:** Perioperative baseline characteristics in living donors undergoing laparoscopic nephrectomy

	Living donors
**n**	**366**
***Preoperative characteristics***	
Sex (male)	154 (42.1%)
Age (years)	46 ± 12
Body mass index ≥25 kg/m^2^	122 (33.3%)
Hypertension	21 (5.7%)
Remnant kidney volume (mL)	176.0 ± 35.4
Estimated glomerular filtration rate (mL/min/1.73 m^2^)	
≥ 90	197 (53.8%)
89–60	169 (46.2%)
*Laboratory variables*	
White blood cell count (× 10^9^/L)	6.1 ± 1.7
Hemoglobin (g/dL)	14.1 ± 1.5
Platelet count (× 10^9^/L)	250.9 ± 58.0
Glucose (mg/dL)	97 ± 10
Albumin (g/dL)	4.4 ± 0.3
Sodium (mEq/L)	142 ± 2
Potassium (mEq/L)	4.3 ± 0.3
Chloride (mEq/L)	105 ± 3
International normalized ratio	1.00 ± 0.06
Activated partial thrombin time (s)	27.7 ± 3.1
***Intraoperative findings***	
Total surgery duration (min)	171 ± 29
Intrathecal morphine block	116 (31.7%)
*Average vital signs*	
Systolic blood pressure (mmHg)	123 ± 13
Diastolic blood pressure (mmHg)	77 ± 9
Heart rate (beats/min)	73 ± 10
Body temperature (°C)	36.4 ± 0.5
Hourly fluid infusion (mL/kg/h)	5.1 ± 2.9
Hourly urine output (mL/kg/h)	1.3 ± 1.1
Total blood loss (mL)	95 ± 98
***Postoperative findings***	
Total days of hospitalization	4 ± 1
Estimated glomerular filtration rate on POD 1	
≥ 60 mL/min/1.73 m^2^	126 (34.4%)
< 60 mL/min/1.73 m^2^	240 (65.6%)
Estimated glomerular filtration rate on POD 7	
≥ 60 mL/min/1.73 m^2^	125 (34.2%)
< 60 mL/min/1.73 m^2^	241 (65.8%)

Values are expressed as means (± SDs) and numbers (percentages)

*Abbreviations*: *POD* postoperative day

### Comparison of pre- and intraoperative findings between living donors with eGFR ≥60 mL/min/1.73 m^2^ and those with eGFR < 60 mL/min/1.73 m^2^ on POD 1

In the preoperative findings (Table 2), living donors with eGFR < 60 mL/min/1.73 m^2^ on POD 1 had a higher proportion of male sex, older age, and higher incidence of hypertension than living donors with eGFR ≥60 mL/min/1.73 m^2^ on POD 1. The laboratory variables revealed that living donors with eGFR < 60 mL/min/1.73 m^2^ on POD 1 had higher hemoglobin and sodium levels, but lower international normalized ratio, compared to living donors with eGFR ≥60 mL/min/1.73 m^2^ on POD 1. Intraoperative findings revealed that living donors with eGFR < 60 mL/min/1.73 m^2^ on POD 1 had a lower proportion of ITMB intervention and lower HR and body temperature levels, compared with living donors with eGFR ≥60 mL/min/1.73 m^2^ on POD 1.

**Table 2:** Comparisons of pre- and intraoperative findings between living donors with eGFR ≥60 mL/min/1.73 m^2^ and those with eGFR < 60 mL/min/1.73 m^2^ on POD 1

Group	eGFR ≥ 60 mL/min/1.73 m^**2**^ on POD 1	eGFR < 60 mL/min/1.73 m^**2**^ on POD 1	***p***
**n**	**126**	**240**	
***Preoperative findings***			
Male sex: n (%)	39 (31.0%)	115 (47.9%)	0.002
Age (years)	43 (29–53)	51 (42–58)	< 0.001
Body mass index ≥25 kg/m^2^	34 (27.0%)	88 (36.7%)	0.062
Hypertension n (%)	2 (1.6%)	19 (7.9%)	0.013
Remnant kidney volume (mL)	173.0 (149.5–203.5)	170.0 (148.5–200.0)	0.717
eGFR			
≥ 90 mL/min/1.73 m^2^	64 (50.8%)	133 (55.4%)	0.399
89–60 mL/min/1.73 m^2^	62 (49.2%)	107 (44.6%)	
*Laboratory variables*			
White blood cell count (× 10^9^/L)	5.9 (5.0–7.0)	5.8 (5.1–6.7)	0.796
Hemoglobin (g/dL)	13.7 (12.8–14.9)	14.1 (13.3–15.3)	0.004
Platelet count (× 10^9^/L)	246.5 (216.8–304.8)	243.0 (213.3–280.8)	0.115
Glucose (mg/dL)	95 (90–101)	97 (92–103)	0.109
Albumin (g/dL)	4.5 (4.3–4.6)	4.5 (4.3–4.6)	0.724
Sodium (mEq/L)	141 (140–142)	142 (141–143)	0.001
Potassium (mEq/L)	4.3 (4.1–4.4)	4.3 (4.1–4.5)	0.519
Chloride (mEq/L)	104 (103–106)	105 (104–106)	0.125
International normalized ratio	1.01 (0.97–1.04)	0.99 (0.96–1.03)	0.010
aPTT (s)	27.4 (26.0–29.1)	27.1 (25.6–28.5)	0.156
***Intraoperative findings***			
Intrathecal morphine block	63 (50.0%)	53 (22.1%)	< 0.001
Total surgery duration (min)	170 (155–190)	170 (147–190)	0.214
*Average vital signs*			
Systolic blood pressure (mmHg)	120 (112–130)	120 (115–130)	0.596
Diastolic blood pressure (mmHg)	80 (70–80)	80 (70–80)	0.298
Heart rate (beats/min)	76 (70–82)	72 (64–80)	< 0.001
Body temperature (°C)	36.5 (36.3–36.8)	36.4 (36.1–36.6)	< 0.001
Hourly fluid infusion (mL/kg/h)	4.7 (3.3–7.0)	4.4 (2.9–6.2)	0.144
Hourly urine output (mL/kg/h)	1.0 (0.7–1.7)	0.9 (0.6–1.6)	0.084
Total blood loss (mL)	50 (50–100)	70 (50–100)	0.353

Values are expressed as medians (interquartile ranges) and numbers (percentages)

*Abbreviations*: *eGFR* estimated glomerular filtration rate, *aPTT* activated partial thrombin time, *POD* postoperative day

### Association of pre- and intraoperative findings with eGFR < 60 mL/min/1.73 m^2^ on POD 1

Multivariable logistic regression analyses (Table 3) suggested that the analgesic intervention of ITMB played a critical and independent role in reducing the potential risk for development of eGFR < 60 mL/min/1.73 m^2^ on POD 1. Additionally, male sex, older age, and a lower hourly fluid infusion rate were significantly associated with a higher risk for development of an eGFR < 60 mL/min/1.73 m^2^ on POD 1. Our risk stratification model for donors with eGFR < 60 mL/min/1.73 m^2^ on POD 1 showed association with non-ITMB, male sex, older age, and lower hourly fluid infusion rate (area under the receiver operating characteristic curve = 0.783; 95% CI = 0.734–0.832; *p* < 0.001).

**Table 3:** Associations of pre- and intraoperative findings with eGFR < 60 mL/min/1.73 m^2^ on postoperative day 1

	Univariable logistic regression analysis				Multivariable logistic regression analysis			
	***ß***	Odds ratio	95% CI	***p***	***ß***	Odds ratio	95% CI	***p***
***Preoperative findings***								
Female sex	−0.719	0.487	0.309–0.768	0.002	− 0.987	0.373	0.214–0.65	0.001
Age (years)	0.055	1.057	1.037–1.077	< 0.001	0.071	1.074	1.05–1.098	< 0.001
Body mass index ≥25 kg/m^2^	− 0.449	0.638	0.398–1.024	0.063				
Hypertension	1.673	5.330	1.221–23.265	0.026				
Remnant kidney volume (mL)	−0.001	0.999	0.993–1.005	0.725				
eGFR ≥90 mL/min/1.73 m^2^	− 0.186	0.83	0.539–1.279	0.399				
*Laboratory variables*								
White blood cell count (× 10^9^/L)	− 0.041	0.960	0.844–1.092	0.535				
Hemoglobin (g/dL)	0.219	1.245	1.072–1.447	0.004				
Platelet count (× 10^9^/L)	−0.005	0.995	0.991–0.999	0.007				
Glucose (mg/dL)	0.012	1.012	0.991–1.035	0.267				
Albumin (g/dL)	−0.286	0.751	0.316–1.786	0.518				
Sodium (mEq/L)	0.205	1.227	1.076–1.399	0.002				
Potassium (mEq/L)	0.396	1.485	0.689–3.202	0.313				
Chloride (mEq/L)	0.079	1.083	0.965–1.215	0.177				
International normalized ratio	−3.633	0.026	0.001–1.172	0.06				
Activated partial thrombin time (s)	−0.054	0.948	0.885–1.015	0.127				
***Intraoperative findings***								
Time effect^a^	0.000	1.000	0.997–1.002	0.639				
Analgesic intervention								
No ITMB	Reference					Reference		
ITMB	−1.261	0.283	0.178–0.451	< 0.001	−1.341	0.262	0.154–0.445	< 0.001
Total surgery duration (min)	−0.004	0.996	0.989–1.003	0.280				
*Average vital signs*								
Systolic blood pressure (mmHg)	0.006	1.006	0.990–1.024	0.458				
Diastolic blood pressure (mmHg)	0.018	1.018	0.994–1.043	0.135				
Heart rate (beats/min)	−0.041	0.960	0.939–0.981	< 0.001				
Body temperature (°C)	−0.765	0.466	0.257–0.843	0.012				
Hourly fluid infusion (mL/kg/h)	−0.065	0.937	0.871–1.009	0.084	− 0.105	0.9	0.826–0.981	0.017
Hourly urine output (mL/kg/h)	− 0.147	0.864	0.714–1.045	0.131				
Total blood loss (mL)	0.000	1.000	0.998–1.003	0.795				

*Abbreviations*: eGFR, estimated glomerular filtration rate; ITMB, intrathecal morphine block

^a^Time effect was determined by the serial order of the living donors from the first (no. 1) to the most recent (no. 366)

In living donors with preoperative eGFRs ≥90 mL/min/1.73 m^2^ (*n* = 197; Additional file 1), preoperative findings of male sex and older age, and several intraoperative findings (i.e., non-ITMB, a higher average DBP, and lower hourly fluid infusion and urine output rates) were associated with a higher risk for development of an eGFR < 60 mL/min/1.73 m^2^ on POD 1. In those with preoperative eGFRs of 89–60 mL/min/1.73 m^2^ (*n* = 169; Additional file 2), a preoperative finding of older age and an intraoperative finding (non-ITMB) were associated with a higher risk for development of an eGFR < 60 mL/min/1.73 m^2^ on POD 1.

### Comparison of pre- and intraoperative findings between the non-ITMB and ITMB groups in PS-matching analysis

Pre- and intraoperative findings in the non-ITMB and ITMB groups were assessed by PS-matching analysis (Table 4). Significant differences were observed in preoperative findings (i.e., sex, an eGFR of 89–60 mL/min/1.73 m^2^, hemoglobin level, and sodium level) and intraoperative findings (i.e., total surgery duration, average DBP and body temperature, hourly fluid infusion rate, and total blood loss), according to ITMB intervention status before PS matching. After PS-matching analysis, no significant differences in pre- or intraoperative findings were observed according to the ITMB intervention.

**Table 4:** Comparisons of pre- and intraoperative findings between non-ITMB and ITMB groups using propensity score-matching analysis

	Before propensity score matched analysis				After propensity score matched analysis			
Group	non-ITMB	ITMB	***p***	SD	non-ITMB	ITMB	***p***	SD
n	250	116			106	106		
***Preoperative findings***								
Female sex (%)	133 (53.2%)	79 (68.1%)	0.007	0.318	65 (61.3%)	70 (66.0%)	0.475	0.101
Age (years)	50 (38–57)	48 (37–55)	0.258	− 0.114	48 (36–56)	48 (37–56)	0.835	0.039
Body mass index ≥25 kg/m^2^	88 (35.2%)	34 (29.3%)	0.266	0.129	32 (30.2%)	33 (31.1%)	0.882	−0.021
Hypertension	18 (7.2%)	3 (2.6%)	0.077	−0.289	2 (1.9%)	3 (2.8%)	> 0.999	0.059
Remnant kidney volume (mL)	170.0 (148.0–202.0)	170.0 (150.0–200.0)	0.938	−0.025	170.0 (148.0–202.0)	170.0 (150.0–200.0)	0.721	0.014
eGFR (89–60 mL/min/1.73 m^2^)	136 (54.4%)	33 (28.4%)	< 0.001	−0.573	41 (38.7%)	33 (31.1%)	0.249	−0.167
*Laboratory variables*								
WBC count (× 10^9^/L)	5.9 (5.0–6.9)	5.8 (5.1–6.8)	0.836	0.009	5.8 (5.0–6.8)	5.9 (5.0–6.8)	0.881	0.012
Hemoglobin (g/dL)	14.1 (13.3–15.3)	13.7 (12.7–14.9)	0.003	−0.323	14.0 (13.2–15.2)	13.8 (12.8–14.9)	0.215	−0.184
Platelet count (× 10^9^/L)	243.0 (211.8–281.8)	248.0 (220.5–294.0)	0.216	0.149	242.5 (215.0–291.3)	243.5 (218.0–294.0)	0.937	−0.029
Glucose (mg/dL)	96 (91–103)	97 (91–103)	0.655	0.083	97 (92–103)	97 (91–102)	0.703	−0.055
Albumin (g/dL)	4.5 (4.2–4.6)	4.5 (4.3–4.7)	0.067	0.245	4.5 (4.3–4.7)	4.5 (4.3–4.7)	0.641	−0.045
Sodium (mEq/L)	142 (141–143)	141 (141–143)	0.024	−0.219	142 (141–143)	142 (141–143)	0.766	−0.028
Potassium (mEq/L)	4.3 (4.1–4.4)	4.3 (4.1–4.5)	0.942	−0.016	4.3 (4.1–4.4)	4.3 (4.1–4.5)	0.57	0.067
Chloride (mEq/L)	105 (104–106)	104 (103–106)	0.090	−0.243	104 (103–106)	105 (103–106)	0.713	−0.133
INR	1.00 (0.96–1.03)	1.00 (0.96–1.04)	0.350	0.096	1.00 (0.97–1.03)	1.00 (0.96–1.04)	0.561	−0.065
aPTT (s)	27.5 (25.6–29.1)	27.0 (25.8–28.2)	0.118	−0.271	27.0 (25.6–28.5)	26.9 (25.8–28.3)	0.686	−0.124
***Intraoperative findings***								
Surgery duration (min)	175 (160–190)	155 (140–180)	< 0.001	−0.531	165 (150–185)	160 (140–185)	0.108	−0.188
*Average of vital signs*								
SBP (mmHg)	120 (116–130)	120 (112–130)	0.727	−0.056	120 (120–130)	121 (112–131)	0.884	0.029
DBP (mmHg)	80 (70–80)	80 (72–88)	0.002	0.339	80 (71–80)	80 (74–87)	0.137	0.158
Heart rate (beats/min)	74 (67–80)	72 (63–80)	0.266	−0.086	74 (64–80)	72 (63–80)	0.455	−0.06
Body temperature (°C)	36.5 (36.3–36.7)	36.3 (36.1–36.6)	0.002	−0.275	36.5 (36.2–36.7)	36.4 (36.1–36.6)	0.311	−0.091
Hourly fluid infusion (mL/kg/h)	4.86 (3.35–7.03)	3.80 (2.56–5.67)	< 0.001	−0.348	4.79 (2.84–6.19)	3.82 (2.62–5.85)	0.215	−0.079
Hourly urine output (mL/kg/h)	0.98 (0.67–1.71)	0.96 (0.63–1.52)	0.261	−0.284	0.86 (0.65–1.39)	0.96 (0.62–1.51)	0.655	0.039
Total blood loss (mL)	95 (50–100)	50 (50–100)	0.017	−0.451	50 (50–100)	50 (50–100)	0.48	−0.068

Values are expressed as median (interquartile) and numbers (proportions)

*Abbreviations*: *ITMB* intrathecal morphine block, *eGFR* estimated glomerular filtration, *WBC* white blood cell, *INR* international normalized ratio, *aPTT* activated partial thrombin time, *SBP* systolic blood pressure, *DBP* diastolic blood pressure

### Comparison of remnant kidney function according to eGFR status on PODs 1 and 7 between PS-matched non-ITMB and ITMB groups

**Fig. 2:**
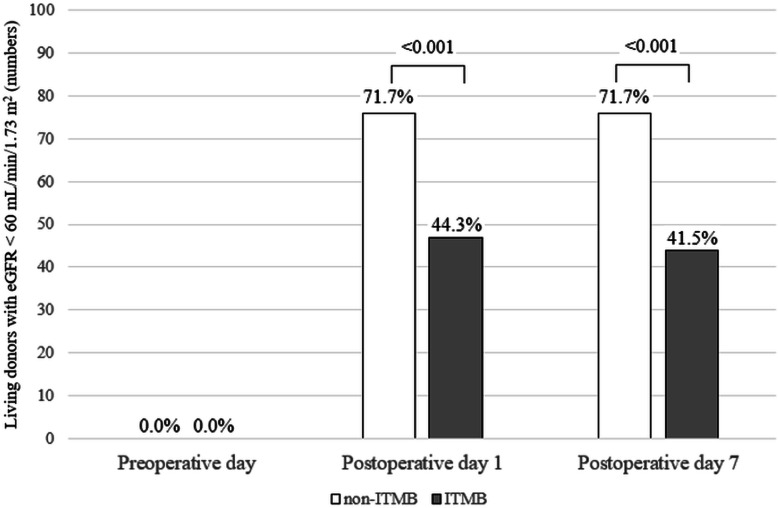
Comparison of remnant kidney function in living donors with eGFRs < 60 mL/min/1.73 m^2^ on the preoperative day and postoperative days 1 and 7 between PS-matched non-ITMB and ITMB groups. Values are expressed as numbers with proportions (%)

The prevalence rates in living donors with eGFR < 60 mL/min/1.73 m^2^ on PODs 1 and 7 were significantly higher in the non-ITMB group than in the ITMB group (Fig. 2).

### After adjustment for PS, ITMB was significantly associated with eGFR < 60 mL/min/1.73 m^2^ on POD 1

After adjustment for PS, the ITMB group was significantly associated with lower risk for development of eGFR < 60 mL/min/1.73 m^2^ on POD 1 in PS-matched living donors (Table 5).

**Table 5:** Association of ITMB with eGFR < 60 mL/min/1.73 m^2^ on postoperative day 1

	***ß***	Odds ratio	95% CI	***p***
**PS-matched living donors (*n*** **= 212)**				
ITMB adjusted for PS	−1.358	0.257	0.14–0.474	< 0.001

*Abbreviations*: *ITMB* intrathecal morphine block, *eGFR* estimated glomerular filtration rate, *PS* propensity score

### Comparisons of postoperative peak NRS and laboratory variables between PS-matched living donors with and without ITMB

On POD 1 (Additional file 3), a high percentage of living donors with ITMB experienced a mild degree of pain (peak NRS ≤3 in 83.0% [*n* = 88] of donors); however, living donors without ITMB generally experienced a severe degree of pain (peak NRS ≥7 in 77.4% [*n* = 82] of donors). Cumulative IV-PCA consumption was higher in the non-ITMB group than in the ITMB group. The peak SBP, DBP and HR values were higher in the non-ITMB group than in the ITMB group.

Laboratory variables (Additional file 4) were comparable between the non-ITMB and ITMB groups on PODs 1 and 7. Although the chloride level on POD 7 differed between the two groups, the levels were within normal limits [27].

During the follow-up period, there were no ITMB-associated complications, such as puncture site infection, post-dural puncture headache, lower limb numbness, respiratory depression, or bleeding, and all living donors were determined to be grade I on the Clavien-Dindo classification.

## Discussion

This study showed that 65.6% (*n* = 240) of living donors undergoing laparoscopic nephrectomy for kidney transplantation exhibited delayed functional recovery of remnant kidney (eGFR < 60 mL/min/1.73 m^2^ on POD 1). Our proposed risk stratification model showed association with preoperative findings (male sex and older age) and intraoperative findings (non-ITMB and lower hourly fluid infusion rate). PS-matching analysis revealed that living donors with ITMB had lower incidences of eGFR < 60 mL/min/1.73 m^2^ on PODs 1 and 7, compared to living donors without ITMB. The analgesic impact of ITMB appeared to lower the risk for delayed functional recovery of remnant kidney (0.257-fold lower than risk in the non-ITMB group) on POD 1.

Although the mechanism connecting analgesia to remnant kidney function remains unclear, good analgesia may safely and effectively enhance remnant kidney function recovery after kidney donation. In our model of risk stratification, ITMB, an analgesic intervention, is clinically modifiable; after PS-matched adjustment, ITMB pain relief attenuated the eGFR loss during the early postoperative period. Effective preoperative pain-relief, such as ITMB, can promote postoperative recovery in patients undergoing abdominal surgery [11]. In patients undergoing aortic valve replacement surgery, ITMB provided appropriate analgesic effects (lower opioid consumption and pain score), hemodynamic stability (tolerable cardiac output), and early postoperative recovery (earlier endotracheal extubation and shorter ICU administration) [28]. In organ transplantation settings, ITMB resulted in predominantly lower pain score on POD 1, compared to other analgesic practices (i.e., IV-PCA, wound infiltration, and peripheral nerve block) [10, 12, 21, 29]. The results of a small KT study by Sener et al. [30] suggested that analgesic care played a role in postoperative organ function recovery, including that of kidneys. However, the authors reported that parameters of kidney graft function (i.e., glomerular filtration rate, microalbuminuria, or creatinine clearance rate) for 2 days postoperatively were similar between grafts from living donors with and without combined spinal-epidural anesthesia. However, a larger KT study by Baar et al. [31] revealed that the incidence of delayed graft function, defined as the requirement of any renal replacement therapy within 1 week postoperatively, was significantly lower in patients who received grafts from living donors with epidural analgesic care than in patients who received grafts from living donors without epidural analgesic care. Potentially, the delayed graft function originates from complex cascades, including hypoxia/ischemia-reperfusion injury and impaired repair mechanisms, which may become aggravated by surgical trauma related to activation of the sympathetic stress response [7, 32]. Therefore, the effective prevention of nociceptive pathways during/after surgery may lead to reduction in overactivity of the sympathetic stress response and subsequent improvement in organ microcirculation and recovery of function [33, 34]. In our study, PS-matched living donors who received ITMB prior to surgery showed markedly improved pain score (i.e., lower peak pain score and cumulative IV-PCA consumption) and more stable hemodynamic parameters (i.e., acceptable SBP, DBP and HR) during the first 24 h postoperatively, compared to those who did not receive ITMB, suggesting that ITMB may attenuate severe pain-related stress responses (i.e., sympathetic activation and vasoconstriction) and maintain homeostasis for optimal function of remnant kidney [35].

In this study, male sex was associated with a higher risk for delayed function recovery of remnant kidney. These findings were supported by Bellini et al. [36], who showed that the reduction in eGFR between pre- and post-donation was greater in male donors, whereas postoperative recovery of kidney function was greater in female donors. Massie et al. [37] reported that male donors had a 1.88-fold greater risk (95% CI = 1.50–2.35; *p* < 0.001) for post-donation renal failure, compared to female donors. Previous studies found that female sex showed stronger protective effects against kidney injury after donor nephrectomy. The authors suggested that sex differences in vulnerability of kidney injury were potentially due to sex hormonal modulation [38–40]. In an experimental model of ischemic kidney injury, rates of severe dysfunction and histologic damage were lower in females than males, and oophorectomy or testosterone administration exacerbated poor renal outcomes. However, estrogen infusion had kidney-protective effects [41, 42]. However, female sex has been regarded as a risk factor for acute kidney injury associated with cardiac surgery, aminoglycoside nephrotoxicity, contrast-induced nephropathy, and rhabdomyolysis [43–46]. The risk to male and female individuals might differ according to the sex-specific impact of factors, such as comorbidities and drug use history.

In this study, older donor age was associated with a higher risk of delayed functional recovery of the remnant kidney. In a European living kidney donor study, younger donors showed a higher post-donation eGFR and better recovery of remnant kidney function compared to older donors (> 60 years) [36]. In a living kidney donor study from the US, older age was also associated with increased risk of delayed functional recovery of the remnant kidney (hazard ratio = 1.40; 95% CI = 1.23–1.59; *p* < 0.001). Donor age is associated with comorbidities of the natural aging process; therefore, the postoperative reserve capacity of kidney function may gradually decrease over time. Nevertheless, the overall safety of living donors at older ages has been acceptable with respect to perioperative outcomes (i.e., operation duration, hemorrhage, hospital admission period, and potential risk for long-term kidney failure) [47, 48]. Additionally, young donors, who are expected to live for more than 60 years, may be more vulnerable to injuries or comorbidities in the future, such as hypertension and DM, compared to older healthy donors and younger individuals who have not undergone organ transplantation [5]. Therefore, a living donation strategy should not be discouraged on the basis of age alone.

In our study, lower hourly fluid infusion rate during surgery was associated with a higher risk for early remnant kidney dysfunction. Clinically, adequate correction of intravascular hypovolemia is a critical component for the prevention and treatment of acute kidney injury [49]. During surgery, optimal maintenance of intravascular volume by intravascular fluid administration is necessary to avoid volume deficiency caused by osmotic loss, evaporation, and hemorrhage. Excessive fluid restriction is associated with an increased risk of organ hypoperfusion and subsequent dysfunction [50–52]. In particular, kidney function is vulnerable to acute changes in volume status, with low volume posing a potential hazard of postoperative kidney damage [49, 53]. Intraoperative pneumoperitoneum during laparoscopic surgery may aggravate the effect of intravascular hypovolemia on systemic circulation, eventually leading to lower renal blood flow and glomerular filtration rate [54]. However, fluid overload is also associated with adverse clinical outcomes, and may directly contribute to kidney injury related to intrarenal compartment syndrome and venous congestion due to encapsulation of the kidneys [55–57]. Aggressive fluid therapy may lead to an imbalance of the renal oxygen supply-demand relationship as a result of increased glomerular filtration rate and sodium reabsorption [58]. Therefore, to maintain appropriate euvolemia, organ perfusion, and oxygen delivery, meticulous monitoring of intraoperative fluid input is essential, in combination with regular estimation of fluid responsiveness and hemodynamic status.

This study had several limitations. First, we were unable to directly measure the analgesic effect of ITMB on systemic/renal hemodynamics. Although our findings regarding severity of pain are consistent with overactivity of the sympathetic stress response, further studies are needed to investigate the role of severe pain on systemic/renal vascular flow and/or perfusion [59, 60]. Second, we were unable to investigate long-term outcomes because of the short analgesic duration of ITMB (within 24 h). However, our donors with ITMB showed better renal recovery in the early (POD 1) and intermediate (POD 7) postoperative periods, compared to donors without ITMB. Because most patients undergoing surgery experience the peak pain level on the first day postoperatively, appropriate and immediate pain relief control may be necessary to achieve enhanced postoperative recovery [61]. Third, we were unable to determine optimal cut-off levels for donor age and hourly fluid infusion. Further prospective studies are needed to investigate such levels for guidance in donation and management. Fourth, we were unable to measure total IV opioid consumption, because various rescue IV opioids were selected based on the preferences and discretion of the attending physicians. The direct effect of IV opioid on remnant kidney function remains unclear; thus, further investigations are needed to determine the role of IV opioid administration, as a component of a multimodal pain-relief approach, on systemic/renal hemodynamics in living donors. Lastly, the ITMB group contained a larger proportion of females, and had a lower BMI and higher baseline eGFR after PS-matching analysis, where all of these factors are associated with improved renal function independent of any effects of ITMB.

## Conclusions

The clinical safety and satisfaction of living donors during the perioperative period are key issues in living donor KT. After nephrectomy, enhanced recovery of remnant kidney function in living donors is critical for preventing the development of chronic kidney dysfunction. This study revealed an association between ITMB and better functional recovery of remnant kidney in living kidney donors. In addition to conferring favorable analgesic results, the use of ITMB resulted in enhanced renal function recovery in living kidney donors. To identify living kidney donors at potential risk for delayed renal function recovery, we propose a stratification model that includes male sex, older age, non-ITMB, and lower hourly fluid infusion rate. Further investigations are needed to confirm our findings in larger populations and in the context of long-term outcomes.

## Supplementary information

**Additional file 1. **Association of pre- and intraoperative findings with eGFR < 60 mL/min/1.73 m^2^ on postoperative day 1 in living donors with preoperative eGFR ≥90 mL/min/1.73 m^2^ (*n* = 197).

**Additional file 2. **Association of pre- and intraoperative findings with eGFR < 60 mL/min/1.73 m^2^ on postoperative day 1 in living donors with preoperative eGFR of 89–60 mL/min/1.73 m^2^ (*n* = 169).

**Additional file 3.** Comparison of pain and hemodynamic outcomes on postoperative day 1 between propensity score-matched living donors with and without intrathecal morphine block.

**Additional file 4.** Comparison of laboratory variables on postoperative days 1 and 7 between propensity score-matched living donors with and without intrathecal morphine block.

## Data Availability

The datasets used and/or analyzed in this study are available from the corresponding author on reasonable request.

## Abbreviations

KT: Kidney transplantation
RRT: Renal replacement therapy
DM: Diabetes mellitus
ITMB: Intrathecal morphine block
IV: Intravenous
SBP: Systolic blood pressure
DBP: Diastolic blood pressure
HR: Heart rate
BT: Body temperature
IV-PCA: Intravenous patient-control analgesia
NRS: Numeric rating scale
PACU: Post-anesthesia care unit
POD: Postoperative day
eGFR: Estimated glomerular filtration rate
BMI: Body mass index
PS: Propensity score
