# Supplementary material for: Delayed remnant kidney function recovery is less observed in living donors who receive an analgesic, intrathecal morphine block in laparoscopic nephrectomy for kidney transplantation: a propensity score-matched analysis
Source: BMC Anesthesiol. 2020 Jul 6;20:165. doi: 10.1186/s12871-020-01081-z (PMC7336465; doi:10.1186/s12871-020-01081-z)
Supplement: Supplementary file 1 — Additional file 1. Association of pre- and intraoperative findings with eGFR < 60 mL/min/1.73 m2 on postoperative day 1 in living donors with preoperative eGFR ≥90 mL/min/1.73 m2 (n = 197). [file 12871_2020_1081_MOESM1_ESM.docx]

**Additional file 1.** Association of pre- and intraoperative findings with eGFR <60 mL/min/1.73 m^2^ on postoperative day 1 in living donors with preoperative eGFR ≥90 mL/min/1.73 m^2^ (n=197)

|  | **Univariable logistic regression analysis** | | | | **Multivariable logistic regression analysis** | | | |
| --- | --- | --- | --- | --- | --- | --- | --- | --- |
|  | ***ß*** | **Odds ratio** | **95% CI** | ***p*** | ***ß*** | **Odds ratio** | **95% CI** | ***p*** |
| ***Preoperative findings*** |  |  |  |  |  |  |  |  |
| Female sex | -1.169 | 0.311 | 0.159 – 0.608 | 0.001 | -1.037 | 0.354 | 0.154 – 0.818 | 0.015 |
| Age (years) | 0.047 | 1.048 | 1.021 – 1.076 | 0.001 | 0.064 | 1.066 | 1.031 – 1.103 | <0.001 |
| Body mass index ≥25 kg/m^2^ | -0.651 | 0.522 | 0.274 – 0.994 | 0.048 |  |  |  |  |
| Hypertension | - | - | - | - |  |  |  |  |
| Remnant kidney volume (mL) | -0.003 | 0.997 | 0.989 – 1.005 | 0.454 |  |  |  |  |
| *Laboratory variables* |  |  |  |  |  |  |  |  |
| White blood cell count (× 10^9^/L) | -0.067 | 0.935 | 0.781 – 1.119 | 0.463 |  |  |  |  |
| Hemoglobin (g/dL) | 0.288 | 1.334 | 1.085 – 1.64 | 0.006 |  |  |  |  |
| Platelet count (× 10^9^/L) | -0.005 | 0.995 | 0.99 – 1.000 | 0.048 |  |  |  |  |
| Glucose (mg/dL) | 0.006 | 1.006 | 0.978 – 1.035 | 0.674 |  |  |  |  |
| Albumin (g/dL) | -0.33 | 0.719 | 0.213 – 2.432 | 0.596 |  |  |  |  |
| Sodium (mEq/L) | 0.177 | 1.194 | 0.997 – 1.429 | 0.054 |  |  |  |  |
| Potassium (mEq/L) | 0.736 | 2.088 | 0.687 – 6.347 | 0.194 |  |  |  |  |
| Chloride (mEq/L) | 0.04 | 1.041 | 0.923 – 1.174 | 0.512 |  |  |  |  |
| International normalized ratio | -4.806 | 0.008 | 0.000 – 2.182 | 0.092 |  |  |  |  |
| Activated partial thrombin time (s) | -0.049 | 0.952 | 0.865 – 1.049 | 0.32 |  |  |  |  |
| ***Intraoperative findings*** |  |  |  |  |  |  |  |  |
| Time effect^†^ | -0.002 | 0.998 | 0.995 – 1.000 | 0.102 |  |  |  |  |
| Analgesic intervention |  |  |  |  |  |  |  |  |
| No ITMB | Reference | | | | Reference | | | |
| ITMB | -1.455 | 0.233 | 0.124 – 0.44 | <0.001 | -1.852 | 0.157 | 0.069 – 0.356 | <0.001 |
| Total surgery duration (min) | -0.008 | 0.992 | 0.982 – 1.002 | 0.119 |  |  |  |  |
| *Average vital signs* |  |  |  |  |  |  |  |  |
| Systolic blood pressure (mmHg) | 0.014 | 1.014 | 0.989 – 1.039 | 0.275 |  |  |  |  |
| Diastolic blood pressure (mmHg) | 0.036 | 1.037 | 1.004 – 1.071 | 0.028 | 0.041 | 1.042 | 1.001 – 1.084 | 0.043 |
| Heart rate (beats/min) | 0.007 | 1.008 | 0.979 – 1.037 | 0.612 |  |  |  |  |
| Body temperature (℃) | 0.154 | 1.166 | 0.681 – 1.997 | 0.576 |  |  |  |  |
| Hourly fluid infusion (mL/kg/h) | -0.114 | 0.892 | 0.811 – 0.983 | 0.021 | -0.139 | 0.87 | 0.771 – 0.981 | 0.023 |
| Hourly urine output (mL/kg/h) | -0.289 | 0.749 | 0.55 – 1.021 | 0.067 | -0.399 | 0.671 | 0.472 – 0.954 | 0.026 |
| Total blood loss (mL) | -0.001 | 0.999 | 0.997 – 1.002 | 0.525 |  |  |  |  |

**Abbreviations:** eGFR, estimated glomerular filtration; ITMB, intrathecal morphine block

^†^A time effect determined by the serial order of the living donors from the first (no. 1) to the most recent (no. 366).
