# Supplementary material for: Delayed remnant kidney function recovery is less observed in living donors who receive an analgesic, intrathecal morphine block in laparoscopic nephrectomy for kidney transplantation: a propensity score-matched analysis
Source: BMC Anesthesiol. 2020 Jul 6;20:165. doi: 10.1186/s12871-020-01081-z (PMC7336465; doi:10.1186/s12871-020-01081-z)
Supplement: Supplementary file 2 — Additional file 2. Association of pre- and intraoperative findings with eGFR < 60 mL/min/1.73 m2 on postoperative day 1 in living donors with preoperative eGFR of 89–60 mL/min/1.73 m2 (n = 169). [file 12871_2020_1081_MOESM2_ESM.docx]

**Additional file 2.** Association of pre- and intraoperative findings with eGFR <60 mL/min/1.73 m^2^ on postoperative day 1 in living donors with preoperative eGFR of 89–60 mL/min/1.73 m^2^ (n=169)

|  | **Univariable logistic regression analysis** | | | | **Multivariable logistic regression analysis** | | | |
| --- | --- | --- | --- | --- | --- | --- | --- | --- |
|  | ***ß*** | **Odds ratio** | **95% CI** | ***p*** | ***ß*** | **Odds ratio** | **95% CI** | ***p*** |
| ***Preoperative findings*** |  |  |  |  |  |  |  |  |
| Female sex | -0.291 | 0.748 | 0.395 – 1.414 | 0.371 |  |  |  |  |
| Age (years) | 0.063 | 1.065 | 1.037 – 1.095 | <0.001 | 0.064 | 1.066 | 1.036 – 1.096 | <0.001 |
| Body mass index ≥25 kg/m^2^ | -0.159 | 0.853 | 0.421 – 1.727 | 0.658 |  |  |  |  |
| Hypertension | 0.386 | 1.471 | 0.277 – 7.816 | 0.651 |  |  |  |  |
| Remnant kidney volume (mL) | 0.002 | 1.002 | 0.993 – 1.011 | 0.726 |  |  |  |  |
| *Laboratory variables* |  |  |  |  |  |  |  |  |
| White blood cell count (× 10^9^/L) | -0.01 | 0.99 | 0.825 – 1.19 | 0.919 |  |  |  |  |
| Hemoglobin (g/dL) | 0.144 | 1.154 | 0.927 – 1.438 | 0.2 |  |  |  |  |
| Platelet count (× 10^9^/L) | -0.006 | 0.994 | 0.989 – 1.000 | 0.06 |  |  |  |  |
| Glucose (mg/dL) | 0.019 | 1.019 | 0.986 – 1.053 | 0.265 |  |  |  |  |
| Albumin (g/dL) | -0.283 | 0.753 | 0.219 – 2.591 | 0.653 |  |  |  |  |
| Sodium (mEq/L) | 0.238 | 1.269 | 1.047 – 1.538 | 0.015 |  |  |  |  |
| Potassium (mEq/L) | 0.000 | 1.000 | 0.34 – 2.94 | 0.999 |  |  |  |  |
| Chloride (mEq/L) | 0.141 | 1.152 | 0.97 – 1.367 | 0.106 |  |  |  |  |
| International normalized ratio | -2.308 | 0.099 | 0.001 – 18.688 | 0.388 |  |  |  |  |
| Activated partial thrombin time (s) | -0.056 | 0.946 | 0.857 – 1.044 | 0.269 |  |  |  |  |
| ***Intraoperative findings*** |  |  |  |  |  |  |  |  |
| Time effect^†^ | 0.001 | 1.001 | 0.998 – 1.004 | 0.44 |  |  |  |  |
| Analgesic intervention |  |  |  |  |  |  |  |  |
| No ITMB | reference | | | | reference | | | |
| ITMB | -1.4 | 0.247 | 0.111 – 0.548 | 0.001 | -1.413 | 0.243 | 0.104 – 0.572 | 0.001 |
| Total surgery duration (min) | 0.001 | 1.001 | 0.99 – 1.013 | 0.805 |  |  |  |  |
| *Average vital signs* |  |  |  |  |  |  |  |  |
| Systolic blood pressure (mmHg) | -0.001 | 0.999 | 0.976 – 1.022 | 0.943 |  |  |  |  |
| Diastolic blood pressure (mmHg) | -0.007 | 0.993 | 0.958 – 1.03 | 0.72 |  |  |  |  |
| Heart rate (beats/min) | -0.016 | 0.984 | 0.954 – 1.016 | 0.328 |  |  |  |  |
| Body temperature (℃) | 0.081 | 1.084 | 0.459 – 2.561 | 0.854 |  |  |  |  |
| Hourly fluid infusion (mL/kg/h) | 0.009 | 1.009 | 0.895 – 1.138 | 0.885 |  |  |  |  |
| Hourly urine output (mL/kg/h) | -0.044 | 0.957 | 0.748 – 1.225 | 0.729 |  |  |  |  |
| Total blood loss (mL) | 0.003 | 1.003 | 0.998 – 1.008 | 0.238 |  |  |  |  |

**Abbreviations:** eGFR, estimated glomerular filtration; ITMB, intrathecal morphine block

^†^A time effect determined by the serial order of the living donors from the first (no. 1) to the most recent (no. 366).
