# Supplementary material for: Delayed remnant kidney function recovery is less observed in living donors who receive an analgesic, intrathecal morphine block in laparoscopic nephrectomy for kidney transplantation: a propensity score-matched analysis
Source: BMC Anesthesiol. 2020 Jul 6;20:165. doi: 10.1186/s12871-020-01081-z (PMC7336465; doi:10.1186/s12871-020-01081-z)
Supplement: Supplementary file 3 — Additional file 3. Comparison of pain and hemodynamic outcomes on postoperative day 1 between propensity score-matched living donors with and without intrathecal morphine block. [file 12871_2020_1081_MOESM3_ESM.docx]

**Additional file 3.** Comparison of pain and hemodynamic outcomes on postoperative day 1 between propensity score-matched living donors with and without intrathecal morphine block

| **Group** | **non-ITMB** | **ITMB** | ***p*** |
| --- | --- | --- | --- |
| **n** | **106** | **106** |  |
| *Pain outcomes* |  |  |  |
| Peak numeric rating scale |  |  | <0.001 |
| 0 - 3 | 1 (0.9%) | 88 (83.0%) |  |
| 4 - 6 | 23 (21.7%) | 17 (16.0%) |  |
| 7 - 10 | 82 (77.4%) | 1 (0.9%) |  |
| Cumulative IV-PCA consumption (mL) | 47.5 (44.0 – 59.5) | 26.0 (24.0 – 29.0) | <0.001 |
| *Hemodynamic outcomes* |  |  |  |
| Peak systolic blood pressure (mmHg) | 144 (134 – 149) | 111 (104 – 114) | <0.001 |
| Peak diastolic blood pressure (mmHg) | 80 (77 – 81) | 70 (64 – 74) | <0.001 |
| Peak heart rate (beats/min) | 91 (89 – 94) | 72 (64 – 78) | <0.001 |

**Abbreviations:** ITMB, intrathecal morphine block; IV-PCA, intravenous patient-control analgesia

**NOTE:** Values are expressed as numbers and proportions.
