# Supplementary material for: Delayed remnant kidney function recovery is less observed in living donors who receive an analgesic, intrathecal morphine block in laparoscopic nephrectomy for kidney transplantation: a propensity score-matched analysis
Source: BMC Anesthesiol. 2020 Jul 6;20:165. doi: 10.1186/s12871-020-01081-z (PMC7336465; doi:10.1186/s12871-020-01081-z)
Supplement: Supplementary file 4 — Additional file 4. Comparison of laboratory variables on postoperative days 1 and 7 between propensity score-matched living donors with and without intrathecal morphine block. [file 12871_2020_1081_MOESM4_ESM.docx]

**Additional file 4.** Comparison of laboratory variables on postoperative days 1 and 7 between propensity score-matched living donors with and without intrathecal morphine block

| **Group** | **non-ITMB** | **ITMB** | ***p*** |
| --- | --- | --- | --- |
| **n** | **106** | **106** |  |
| *Laboratory variable on postoperative day 1* |  |  |  |
| White blood cell count (x 10^9^/L) | 10.0 (8.3 – 11.7) | 10.4 (9.0 – 12.1) | 0.213 |
| Hemoglobin (g/dL) | 11.4 (10.8 – 12.9) | 11.6 (10.9 – 12.6) | 0.55 |
| Platelet count (x 10^9^/L) | 195.0 (166.0 – 238.0) | 200.0 (171.3 – 234.3) | 0.566 |
| Sodium (mEq/L) | 138 (137 – 140) | 139 (137 – 140) | 0.087 |
| Potassium (mEq/L) | 3.9 (3.7 – 4.1) | 3.9 (3.8 – 4.2) | 0.287 |
| Chloride (mEq/L) | 104 (103 – 106) | 104 (103 – 106) | 0.792 |
| *Laboratory variable on postoperative day 7* |  |  |  |
| White blood cell count (x 10^9^/L) | 7.0 (6.1 – 8.4) | 7.2 (6.2 – 8.8) | 0.407 |
| Hemoglobin (g/dL) | 13.4 (12.2 – 14.5) | 13.3 (12.4 – 14.3) | 0.92 |
| Platelet count (x 10^9^/L) | 309.0 (263.8 – 368.3) | 333.0 (277.5 – 377.0) | 0.159 |
| Sodium (mEq/L) | 140 (139 – 141) | 140 (138 – 141) | 0.056 |
| Potassium (mEq/L) | 4.6 (4.4 – 4.8) | 4.6 (4.4 – 4.9) | 0.706 |
| Chloride (mEq/L) | 102 (101 – 104) | 101 (100 – 103) | 0.023 |

**Abbreviations:** ITMB, intrathecal morphine block

**NOTE:** Values are expressed as median (interquartile) and numbers (proportions).
